# Combination of Q-switched 1,064 and 532 nm Nd: YAG laser in the treatment of toenail onychomycosis: a pilot study

**DOI:** 10.1007/s10103-025-04712-4

**Published:** 2025-10-25

**Authors:** Clara Gómez, Enrique Alberdi

**Affiliations:** 1 Instituto de Química Física Blas Cabrera, CSIC, Madrid, Spain; 2 Private Clinic of Dr. Alberdi, Madrid, Spain

**Keywords:** Q-switched Nd:YAG 1,064 nm/532 nm laser, Toenail onychomycosis

## Abstract

Previous studies have demonstrated that laser-based therapies can effectively improve the appearance of nails affected by onychomycosis, offering a high safety margin, making them suitable for patients in whom systemic antifungal agents are contraindicated. Building on earlier studies, a pilot clinical trial was designed to assess the effectiveness of a Q-switched Nd: YAG laser using dual wavelengths (1,064 nm and 532 nm) for the treatment of toenail onychomycosis. This study involved 15 participants with distal lateral subungual onychomycosis (DLSO) of the big toe caused by dermatophytes. Each laser session included two applications of 1,064 Nd: YAG laser at 600 mJ/pulse, 20 ns, 2 Hz, for 1 min, separated by a 1-minute pause. After a 2-minute rest, the 532 nm laser was applied in a similar manner using the same parameters. Treatments were administered weekly over eight weeks. Progress was monitored using the Onychomycosis Severity Index (OSI), based on direct clinical examination and photographs taken at baseline, during each session, and at follow-up visits. Mycological clearance was assessed via PAS-stained histopathology and fungal culture. Pain during treatment was evaluated using a visual analogue scale (VAS), and any side effects were recorded. At week 43, both mycological cure rate (MCR) and complete cure rate (CCR) were 33.3% (5 out 15 patients). The laser therapy was well-tolerated, with no reported adverse effects. Pain scores averaged 6.5 ± 0.5 during the 1,064 nm application and 7.8 ± 0.5 during the 532 nm application. Despite some discomfort, patient satisfaction remained high. The dual-wavelength Q-switched Nd: YAG laser appears to be a promising treatment option for mild onychomycosis, although its effectiveness is limited in severe onychomycosis. Further research is needed to optimize laser parameters, session frequency, and follow-up duration to maximize therapeutic outcomes.

## Introduction

Onychomycosis is a chronic fungal infection of the nails caused by dermatophytes, yeasts, and non-dermatophyte molds [1]. It is the most common nail infection encountered in clinical practice, accounting for about 30% of all cutaneous fungal infections, 50% of all onychopathies, and about 90% of toenail infections worldwide [2]. Although many patients are primarily affected by cosmetic issues, nail abnormalities caused by onychomycosis can also result in emotional distress and functional impairments, which can reduce overall quality of life [3].

The treatment of onychomycosis presents a challenge for dermatologists and typically involves nail debridement, along with topical and systemic antifungal therapies. Oral antifungals may be associated with various side effects, while topical therapy is often ineffective when used as a monotherapy [4].

The introduction of laser technology has brought significant changes to dermatological treatments, including those for onychomycosis. Non-pharmacological alternatives aimed at improving the clearance of onychomycosis include long-pulsed and Q-switched Neodymium-doped Yttrium Aluminium Garnet (Nd: YAG) lasers, photodynamic therapy, near-infrared diode lasers, and fractional CO_2_ lasers [5]. In 2011, the U.S. Food and Drug Administration (FDA) approved Nd: YAG laser devices as a method to temporarily improve the appearance of affected nails. Since then, a growing number of studies have been published on this topic [6].

Laser therapy using wavelengths between 750 and 1300 nm exerts antifungal action through several mechanisms [7]. First, fungal hyphae contain chromophores that selectively absorb laser energy, which is then converted into heat. This heat denaturalizes essential proteins and cellular components, thereby inhibiting fungal growth [8]. Second, laser energy can physically disrupt treatment-resistant biofilms, improving the effectiveness of antifungal drugs or deeper laser penetration [9]. Third, the 1,064 nm Nd: YAG laser can penetrate deeply into tissue, generating localized heat that stresses fungal cells. This stress triggers the formation of reactive oxygen species (ROS), leading to oxidative damage to DNA and organelles and initiating cell death pathways [10]. Additionally, effective laser treatment depends on the transfer of heat to fungal mycelium, raising temperatures to a fungicidal range of 43–51 °C for 2–3 min. This thermal effect may also improve local blood flow and stimulate the immune response [11].

Q-switched lasers operate on the principle of selective photothermolysis, in which laser energy is preferentially absorbed by the fungal mycelium. This results in a rapid increase in temperature within the fungal structures causing their destruction [12]. Because the treatment is highly targeted, the surrounding tissue remains unaffected, minimizing the risk of systemic side effects [13]. In this context, lasers should have a wavelength between 750 and 1300 nm to ensure adequate nail penetration, a shorter pulse duration than the fungus’s thermal relaxation time, and a uniform beam to avoid the formation of “hot spots” [5]. Although Q-switched 532 nm Nd: YAG laser does not penetrate the nail as deeply as the Q-switched 1,064 nm Nd: YAG laser, it is selectively absorbed by xanthomegnin [14]. Based on preliminary studies, a clinical trial was conducted to assess the antifungal and clinical effectiveness of a dual-wavelength (1,064 and 532 nm) Q-switched Nd: YAG laser as monotherapy for the treatment of toenail onychomycosis.

## Methods

Fifteen adult patients with a clinical diagnosis of distal-lateral subungual onychomycosis (DLSO) affecting a single nail of one of the two first toes were recruited (Table 1). The diagnosis was confirmed via nail biopsy, which was histopathologically examined using periodic acid-Schiff (PAS) staining and fungal culture. Patients were recruited from the private clinic of Dr. Alberdi between April and September 2024. Potential participants were screened for eligibility according to predetermined inclusion and exclusion criteria. The inclusion criteria were: age over 18 years, confirmed presence of hyphae in the nail sample, a diagnosis of DSLO, dermatophyte infection limited to no more than one nail per patient, and overall good health as assessed by a physician. The exclusion criteria were: subjects with complete fungal involvement of the nail, proximal subungual onychomycosis, severe secondary dermal infection or damaged or broken periungual skin, nail psoriasis, history of peripheral arteria disease, diabetes mellitus, peripheral neuropathy, subjects on immunosuppressive drugs or with medical conditions that increase the risk of infection or delay wound healing, and subjects who have used oral or systemic antifungal therapies in the last six months.

**Table 1:** Characteristics of the recruited patients and baseline conditions of the selected nails

CHARACTERISTICS	
Age [mean±(SD)];	58.0 ± 12.2;
range; median	[40, 69]; 66
Sex	
Male [n (%)]	10 (66.7%)
Female [n (%)]	5 (33.3%)
Culture reports [n (%)]	
T rubrum	12 (80%)
T interdigitale	3 (20%)
OSI [mean±(SD)]	18.6 ± 8.6
Severity of the disease [n (%)]	
Mild	1 (6.7%)
Moderate	5 (33.3%)
Severe	9 (60%)
Area affected (%) [mean±(SD)]	50.3 ± 25.7
Chronicity (year)[mean±(SD)]	2.9 ± 1.5

n, number of patients; %, percentage of patients; SD, standard deviation

**Fig. 1:**
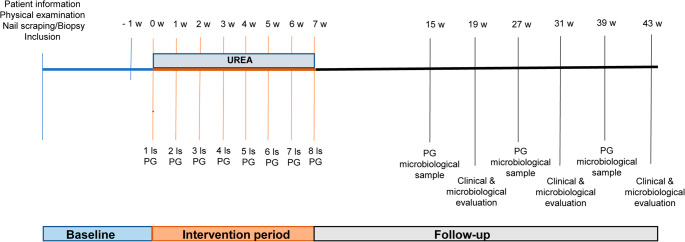
Graphical representation of the study protocol, specifying the experimental and follow-up periods (including clinical and microbiological evaluations). PG: photographs, w: week, ls: laser session

Patients were informed about the laser treatment, its potential side effects and available therapeutic options, after which they provided written informed consent. Each patient signed a consent form and authorized clinical photography of their nails before and after laser therapy. The study was conducted with the approval from local ethics committee and was performed in accordance with the Declaration of Helsinki [15]. A diagram outlining the study protocol and corresponding timeframes is presented in Fig. 1.

### Treatment protocol

Prior to laser treatment, the nail was softened with urea to facilitate the penetration of laser radiation through the nail plate. A compounded topical formulation containing 40% urea, obtained from a pharmacy, was applied to the affected nail area and covered with a fabric adhesive tape each night for 12 h, for five consecutive days prior to each laser irradiation session.

Laser treatment was performed using a Q-switched 1,064/532 nm Nd: YAG laser from ALMA Lasers. The treatment protocol was as follows: first, the Nd: YAG radiation at 1,064 nm with a pulse duration of 20 ns was set to a fluence of 600 mJ/pulse over a 3-mm spot at 2 Hz and applied for 1 min. After a 1-minute pause, the affected nail area was irradiated again for 1 min using the same parameters. Following a 2-minute pause, Nd: YAG radiation at 532 nm with a pulse duration of 20 ns was applied, with the fluence also adjusted to 600 mJ/pulse over a 3-mm spot at 2 Hz. Finally, after a 1-minute break, the affected nail area was irradiated with Nd: YAG at 532 nm, using the same parameters. A total of eight laser sessions were performed, spaced over the course of one week.

### Assessment of therapeutic endpoints

The clinical progression of the nails was objectively evaluated using the Onychomycosis Severity Index (OSI), which allows categorization into mild, moderate or severe onychomycosis, facilitates monitoring of disease progression, and enables comparisons with data from other studies using the same index [16]. OSI data were obtained through direct observation of the nails, since accurately assessing thickness from photographs is very challenging. All measurements - both OSI and percentage of nail involvement - were consistently performed by the same highly experienced investigator. OSI scores were recorded at baseline, at the end of treatment sessions, and during follow-ups visits. The percentage of clinical improvement was estimated from the recorded OSI scores [17]. Progression or regression of the nail’s clinical condition was also documented through a series of photographs taken throughout the study. A CANON EOS 1000D digital single-lens reflex camera with a 10.1-megapixel image sensor was used under standardized lighting and distance conditions, allowing for consistent monitoring and documentation of visual changes.

Mycological evaluation was done on a nail clipping sample taken from the distal edges of the affected toenails for histopathological examination with a periodic acid-Schiff stain, complemented by microbial culture analysis of the material obtained from scraping the inner part of the nail bed. Mycological remission was defined as the absence of pathogens in both histopathological examination and fungal culture. Complete cure was considered achieved when there was a negative culture for dermatophytes, negative PAS staining, with no residual clinical signs of the disease or a marked clinical improvement of the target toenail. Side effects (e.g., redness, oedema, burning, blisters…) were recorded using a survey collected prior to each laser session. Pain during each laser session was measured using a visual analogue scale (VAS), based on the question: “How intense is your pain right now?” with a scale ranging from 0 (no pain) to 10 (most intense pain).

### Statistical analysis

Statistical evaluation was performed using the SPSS software package for Windows (version 30; SPSS, Chicago, III). The assumption of normality was corroborated with the Shapiro-Wilk test. Quantitative data were presented as mean values with standard deviations, while qualitative data are expressed as frequencies and percentages. To evaluate changes in OSI, percentage of nail involved, and degree of clinical improvement values over time (baseline, 19, 31, 43 weeks), a General Linear Model (GLM) with repeated measures was used. To assess changes in mycological cure rates (MCR) and complete clinical rate (CCR) over time, the Related-Samples Cochran´s Q test was used. A p-value below 0.05 was considered statistically significant.

## Results

Nineteen patients were enrolled and treated. Four patients were excluded due to failure to attend follow-up visits, resulting in 15 patients completing the treatment period and follow-up evaluations.

The reduction in both OSI values and the affected nail area over the evaluation period provides evidence of the effectiveness of the laser treatment (Table 2), particularly up to week 19, after which improvement plateaued and remained stable through week 43. A significant reduction is OSI values was observed between baseline and weeks 19, 31 and 43 (*p* < 0.05). Similarly, a significant reduction in the percentage of involved nail area was detected between baseline and weeks 19, 31 and 43 (*p* < 0.05). However, no significant differences were found in the percentage of clinical improvement between week 19 and week 31, nor between week 31 and week 43 (Table 2).

**Table 2:** Evolution of the OSI, degree of clinical improvement, and percentage of nail involvement throughout the study

OSI [mean±(SD)]	
Baseline	18.6 ± 8.6
19-week follow-up	10.3 ± 6.6^a^
31-week follow-up	10.8 ± 6.8^a^
43-week follow-up	10.9 ± 7.7^a^
Degree of improvement Week 19	
No	6
Mild	1
Moderate	6
Severe	2
*Average value*	46.3 ± 35.5
Degree of improvement Week 31	
No	5
Mild	4
Moderate	2
Severe	4
*Average value*	44.5 ± 34.7
Degree of improvement Week 43	
No	7
Mild	3
Moderate	0
Severe	5
*Average value*	44.7 ± 38.5
Area affected (%) [mean±(SD)]	
*Baseline*	50.3 ± 25.7
*19-week follow-up*	29.7 ± 15.9^a^
*31-week follow-up*	32.2 ± 18.0^a^
*43-week follow-up*	33.3 ± 24.9^a^

^a^ The value is statistically significantly different compared to the baseline value

The percentage of clinical improvement remained stable across the follow-up periods (weeks 19, 31, and 43), as improvements observed in some nails were offset by relapses in others, resulting in a consistent overall value (Table 2).

**Fig. 2:**
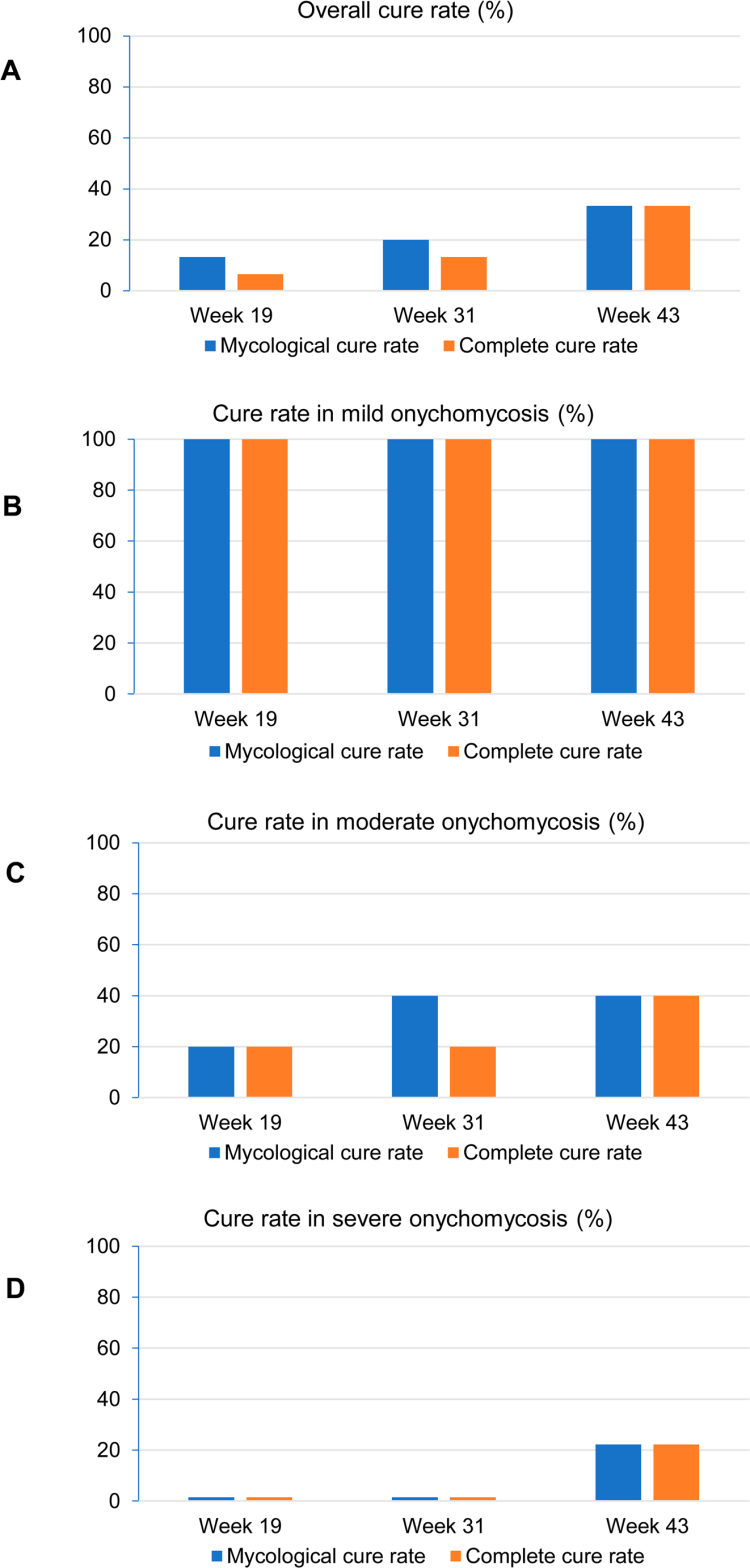
Evolution of the mycological and complete cure rates based on disease severity. (**A**) overall, (**B**) mild severity, (**C**) moderate severity and (**D**) marked severity

**Fig. 3:**
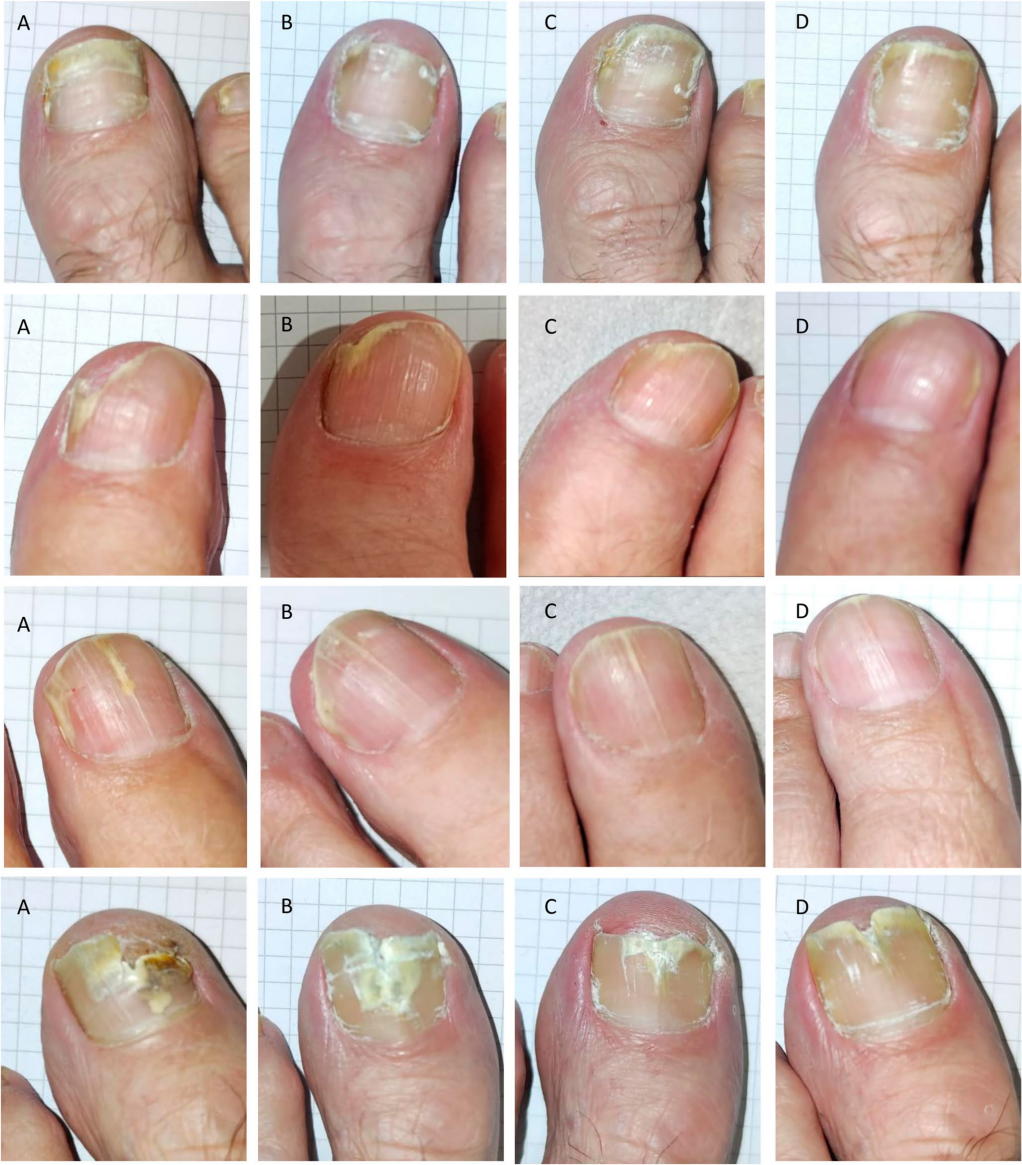
Longitudinal follow-up of onychomycosis. (**A**) Baseline. (**B**) Nail appearance after 19 weeks of treatment, (**C**) after 31 weeks, and (**D**) after 43 weeks

The overall mycological and complete cure rate (MCR and CCR) were 33.3% at week 43, being this value statistically significant compared to the baseline (*p* < 0.05). At this point, the CCR for mild cases was 100% (a level already reached by week 19); for moderately severe cases, the CCR was 40%, whereas only 22.2% of patients with marked severity showed evidence of cure (Fig. 2). A clear inverse relationship was observed between disease severity and treatment response. A selection of cases exhibiting a favorable response to the treatment over the entire evaluation period is presented in Fig. 3.

Nd: YAG laser therapy at both 1,064 nm and 532 nm wavelengths proved to be safe. No complications or adverse effects were reported with this treatment modality. No topical anesthesia was required prior to irradiation. Patients reported a mean pain score of 6.5 ± 0.5 during 1,064 nm sessions and 7.8 ± 0.5 during 532 nm sessions, although they expressed overall satisfaction with the treatment throughout the study. 

## Discussion

The primary benefits of laser treatment for onychomycosis over conventional oral or topical antifungal medications include its simplicity of use, minimal contraindications, and a general lack of side effects. However, previous studies carried out employing laser radiation for onychomycosis treatment have demonstrated limited efficacy, and thus its use has generally been restricted to a select group of patients for whom standard oral therapy is not feasible [18]. The Q-switched Nd: YAG laser generates high-energy peaks with many repetitions, and thus, it does not warm the tissue and produces impact energy that mechanically damages only the target of interest (fungi). The 532 nm wavelength is thought to more effectively target the fungal pigment xanthomegnin, whereas the 1,064 nm is preferably absorbed by components of the fungal cell wall [19].

Among the studies reporting favorable outcomes with the use of these devices, two are particularly noteworthy. The first, conducted using a dual-wavelength Q-switched Nd: YAG laser as monotherapy on 131 subjects with onychomycosis, reported a clinical and MCR of 95.42% [20]. The second study, which also utilized a Q-switched Nd: YAG laser (1,064 nm/532 nm), reported 100% MCR three months after treatment [21].

The results presented in the current study indicate a CCR of 33.3% at 43 weeks. Although this rate is modest, it reflects the therapeutic potential of both laser radiations. In this study, a single cycle of 8 treatment sessions was applied. Other studies have shown that administering 12 sessions in two cycles (8 and 4 sessions) yields higher MCRs [22]. Probably, choosing the best profile in patients and setting the best energy density and number of sessions may further enhance response rates in the future. Additionally, by focusing on participants with involvement limited to a single toenail, our study enhanced the precision and reliability of its findings, serving as a preliminary approach to identify factors that may influence treatment outcomes [23].

Urea at concentrations above 30% acts by softening and hydrating the nail plate through keratinolysis, breaking down the keratin structure to improve the absorption and bioavailability of topical antifungal agents [24]. The laser’s heat and its ability to disrupt fungal biofilms further contribute to infection clearance. Combined, the chemical effects of urea and the physical impact of laser treatment offer a well-rounded antifungal strategy, targeting both the fungal organisms and the protective barriers of the nail. In our study, laser treatment was combined with urea pretreatment under occlusion to enhance eficacy, an approach previously validated by Cai et al., albeit using a long-pulsed Nd: YAG laser [25]. Nevertheless, the MCR and CCR values obtained here remain low compared to those achieved with other light-based therapies combined with urea for the treatment of onychomycosis [26–28].

Since short-pulsed 1,064 nm-Nd: YAG laser received FDA approval in 2010 for the treatment of onychomycosis, its role has been remained a topic of ongoing debate [29]. Current research has not provided conclusive evidence supporting its efficacy (whether in long-pulsed mode or Q-switched modes) from an evidence-based medicine perspective. Whereas some authors have reported favourable outcomes using long-pulsed [22] or Q-switched [20, 21] laser modes, others have found limited or unsatisfactory responses [29, 30]. The lack of valid conclusions is largely attributable to methodological limitations, including short follow-up periods used, inadequate mycological diagnostics, recruitment of a short and inhomogeneous study population, and variability in onychomycosis types and causative fungal species [18, 29]. One potential avenue for future research is to investigate whether combining long-pulsed and Q-switched Nd: YAG laser modalities within a single treatment protocol could enhance the overall effectiveness of this laser radiation for onychomycosis.

The results of the present study also demonstrated that, after 19 weeks, some patients (particularly those with severe onychomycosis) exhibited no further improvement in OSI score or in the percentage of affected nail area, suggesting a low efficacy of the treatment and its insufficiency in preventing relapses. Our study found that the effectiveness of Nd: YAG laser therapy for treating onychomycosis is relatively low when compared to the efficacy rates of oral antifungal treatments [18] or other light-based therapies [27, 28].

A standardized laser treatment protocol for onychomycosis has not yet been established. Various laser devices have been utilized, differing in pulse duration, number of treatment sessions, and the time intervals between sessions, with results diverging from clinical inefficiency to high mycological cure rates. What is being observed is that the higher are the energy density and the number of sessions, the higher will be the clinical and the mycological cure rates [31]. In addition, it was suggested that the therapeutic response to 1,064 nm Nd: YAG laser may be influenced by the clinical type of onychomycosis. Superficial white onychomycosis (SWO) has shown better clinical response compared to DLSO [31]. The present study focused exclusively on nails affected by DLSO, which may partially explain the modest efficacy observed. Initial severity of infection also appears to impact on therapeutic response [32]. In our observations, the response to laser therapy was very good in mild cases, acceptable in moderate cases, and limited in severe cases. Increasing evidence supports that in cases of severe onychomycosis, the effectiveness of Nd: YAG laser therapy is significantly reduced. Therefore, its use should be considered in combination with topical or systemic antifungal agents in such cases [33]. In addition, no different sensitivities to laser radiation were observed between the two fungal species detected in the nails of the selected patients. Study limitations include a small sample size with a high proportion of patients with severe onycomycosis, lack of a control group, and the focus on treating only dermatophytic onychomycosis, which may exhibit lower resistance compared to other fungal pathogens responsible for onychomycosis. Nevertheless, it provides valuable insights into the use of combined Q-switched Nd: YAG laser radiation in the treatment of DLSO. Specifically, it highlights the limited efficacy of the treatment in severe cases and its potential usefulness primarily in mild forms of the disease. It is essential to define optimal laser settings and parameters - including the number of sessions, intervals between sessions, and appropriate follow-up duration - to achieve the best therapeutic outcomes in the treatment of mild DLSO onychomycosis.

## Data Availability

The datasets generated during and/or analyzed during the current study are available from the corresponding author on reasonable request.
